# Multimodal approach in a patient with Fournier's gangrene during the coronavirus pandemic

**DOI:** 10.1016/j.eucr.2021.101825

**Published:** 2021-08-28

**Authors:** Francesco Trama, Ester Illiano, Giancarlo Bertuzzi, Stefano Chiummariello, Elisabetta Costantini

**Affiliations:** a Andrology and Urogynecology Clinic, Santa Maria Terni Hospital, University of Perugia, Italy; b Plastic Surgery Clinic, Santa Maria Terni Hospital, Terni, Italy

**Keywords:** Fasciitis, Necrotizing, Fournier's gangrene, COVID-19

## Abstract

Fournier's gangrene (FG) is a polymicrobial necrotizing fasciitis involving the external genitalia and perineal region. It preferentially affects men, with a mortality rate of approximately 40%. Early diagnosis, surgical debridement, appropriate antibiotic therapy, and hyperbaric chamber treatment increase the therapeutic success. The purpose of this clinical report was to emphasize how a multimodal treatment and the tenacity of the health professionals involved in the era of the coronavirus pandemic with considerable health and logistical difficulties can lead to the complete recovery of patients suffering from FG.

## 1 Introduction

Fournier's gangrene (FG) is a polymicrobial, fulminant, and rare form of necrotizing fasciitis that affects the perineal, genital, and perianal areas. Men were affected at a 10:1 ratio compared to women. Predisposing factors include atherosclerosis, diabetes mellitus, alcoholism, peripheral arterial disease, and immunosuppression.^1^

The bacterial species responsible are both aerobic and anaerobic bacteria, particularly *Streptococcus* spp.*, Enterococcus* spp.*, and Bacteroides* spp. Bacterial infection, favored by some conditions, such as diabetes, develops thrombosis of small vessels with reduced oxygen supply in the tissues, which generates a proliferation of anaerobic bacteria. Moreover, lytic enzymes are produced by bacteria that destroy tissues and cause altered leukocyte activity with the impossibility to fight the infection.^2^

The diagnosis is purely clinical and is based on the symptoms that the patient presents with scrotal edema, erythema, scrotal pain, cutaneous and subcutaneous necrosis with tissue crepitations, feculent odor, and fever.^1^

The treatment consists of aggressive and early debridement of the involved necrotic tissue, empiric antibiotic therapy, and then, according to the results of cultures and use of hyperbaric oxygen therapy (HBOT).^3^

The aim of this clinical case is to emphasize the need for an early multimodal approach to the FG patient and to ensure the best evidence-based care available despite the difficulties caused by SARS-CoV-2 infection.

## 2 Case presentation

A 56-year-old man was presented to our emergency department in December 2020. The patient reported scrotal pain with itching and difficulty in urination. The patient reported that due to the ongoing pandemic in Italy with thousands of cases of SARS-CoV-2 infection and fear of contracting the infection, he had not visited the hospital or his family doctor earlier. The family doctor had prescribed ceftriaxone 1 g/day intramuscular (IM) for 7 days and non-steroidal anti-inflammatory drugs (NSAIDs) as needed over the telephone approximately 10 days earlier.

The patient had the following blood chemistry values at admission to the hospital: Hb 9.2 g/dL, leukocytes 21.17 × 103/mm^3^, neutrophils 13.79 × 103/mm^3^ (80.3%), glucose 373 mg/dL, Hb glycated 11.5%, serum sodium 151 mmol/L, serum potassium 3.1 mmol/L, hematocrit 39.3%, serum creatinine 0.7 mg/dL, serum bicarbonate 33 mmol/L. In addition, the skin temperature was 38.3 °C, heart rate was 182 bpm, and respiratory rate was 33 breaths per minute.

The FG severity index was 11.^4^

The patient had chronic ischemic heart disease about 12 months earlier, a smoker, BMI of 29.5 kg/m^2^. In addition, family members reported that the subject had been an alcoholic for five 5 years.

**Fig. 1:**
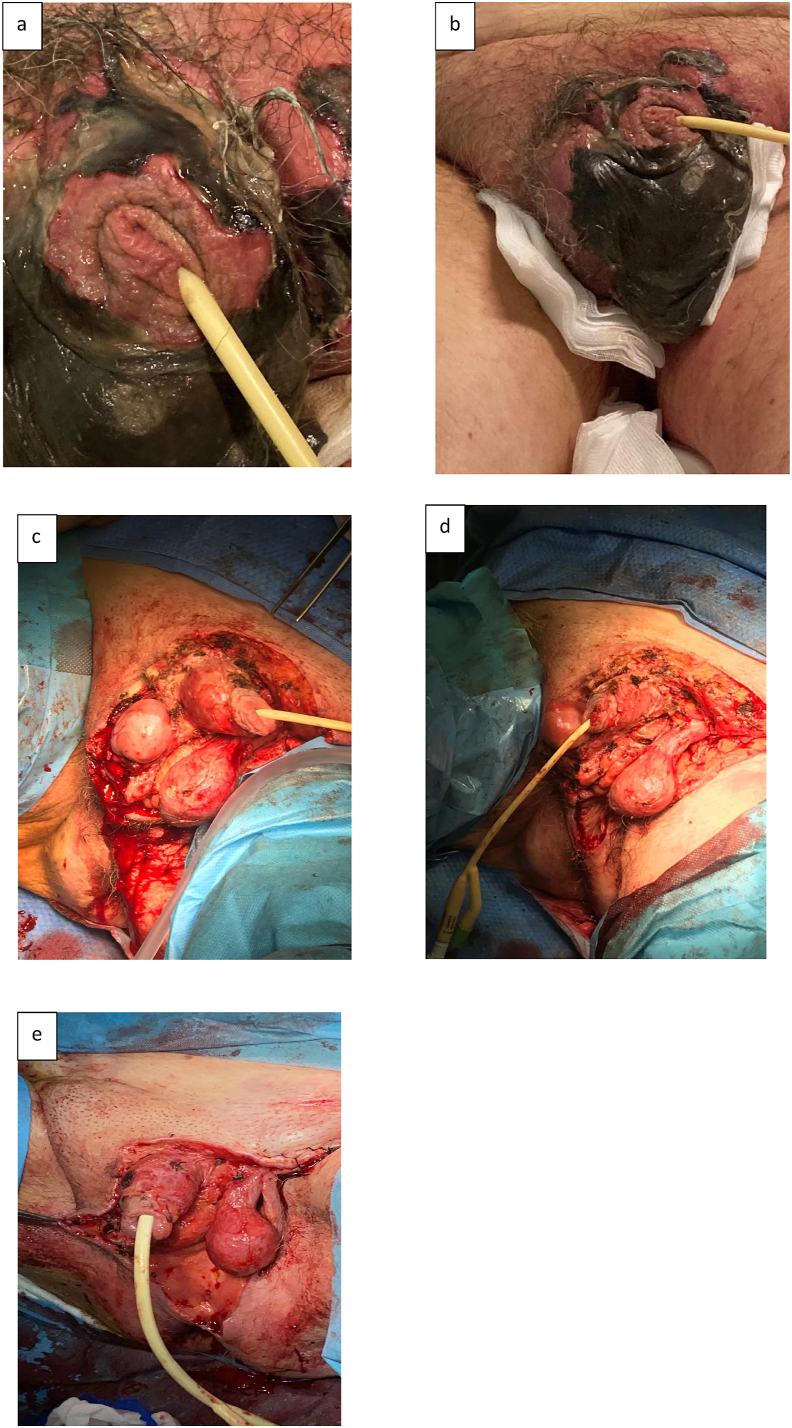
(a–b) Clinical inspection of the patient at the emergency department; (c–e): surgical debridement of necrotic tissues and concomitant right orchiectomy.

On inspection, there was a frankly necrotic area involving the perineal, penile, scrotal region, and partial external anal sphincter with the formation of a perineal-anal fistula (Fig. 1 a – b). A characteristic fecaloid odor was present. On palpation, subcutaneous tissue crepitations and intense pain were observed (VAS 8/10).

The patient was hemodynamically stabilized with intravenous fluid infusion and broad-spectrum empiric parenteral antibiotic therapy including metronidazole, gentamicin, and third-generation cephalosporin. He was immediately taken to the operating room where aggressive debridement of the necrotic tissues was performed, the anal perineal fistula was repaired, and a right orchidectomy was performed because of necrosis involving the didyme itself (Fig. 1 c – e).

At the time of surgery, swabs were performed and necrotic tissue was collected and used for microbiological analysis according to the European Committee on Antimicrobial Susceptibility Testing guidelines.

Subsequently, once the results of the microbiological analysis were obtained (*Escherichia coli and Bacteroides caccae* were identified) an adjustment was made to the antibiotics taken by the patient and the 3rd generation cephalosporin was replaced with a carbapenem for 30 days.

The urologists on the day after the surgery contacted several facilities specializing in hyperbaric treatment. Unfortunately, due to the pandemic, the staff had been relocated to other locations. To safeguard the patient's life and quality of life, several other hospitals, even hundreds of kilometers away; were contacted, and, finally, a facility 122 km away was found that could accept the patient for treatment but not for hospitalization due to the scarcity of beds.

It was then decided to transport the patient every day by ambulance with specialized nursing staff for the duration of the treatment and to ensure that the patient could undergo the hyperbaric treatment. HBOT was performed at 100% oxygen at 2.5 absolute atmospheres (2.5 ATA) of pressure for 100 minutes for 21 days from Monday to Friday. The patient did not incur the health costs for this treatment thanks to the Italian national health system that protects the right to health.

**Fig. 2:**
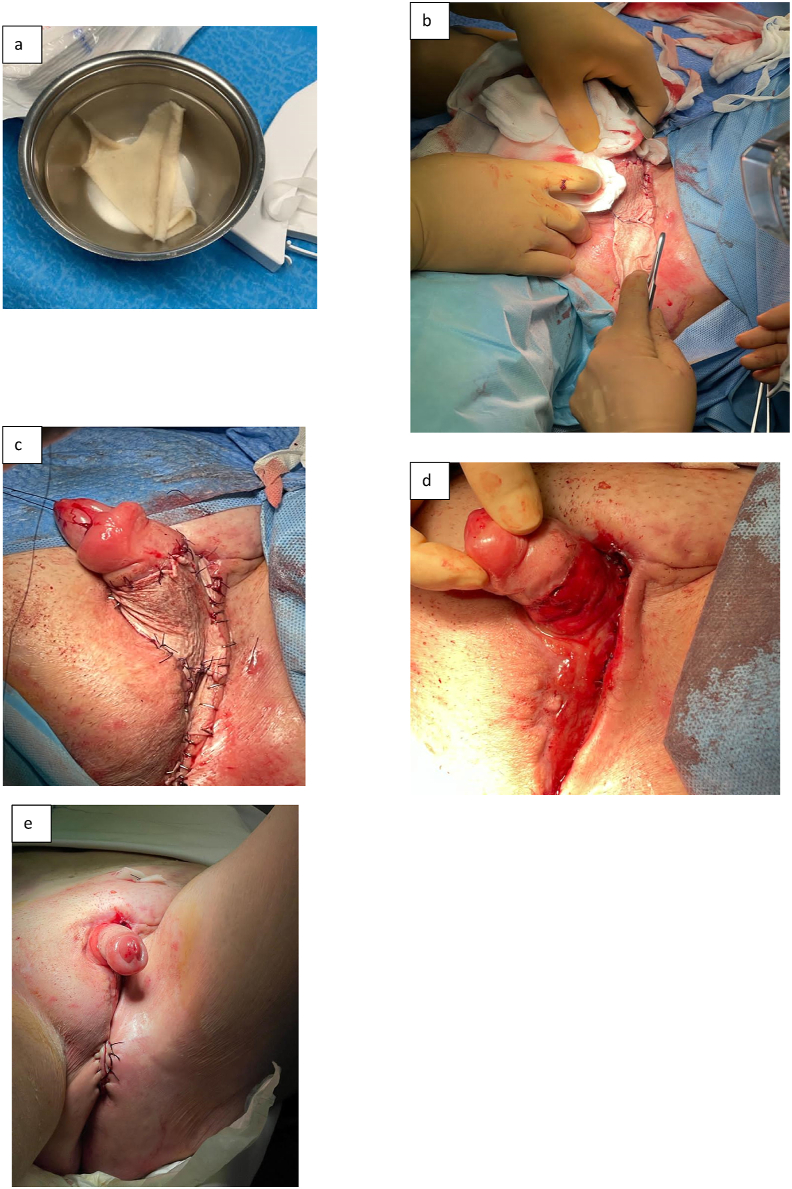
(a) Skin graft obtained from the right thigh; (b–e): reconstructive surgery of perineal, scrotal, penile and suprapubic skin with the previously isolated skin graft.

Once the partial restoration of the necrotic tissues with new granulation tissue was obtained, together with the plastic surgeons, we proceeded to perform skin sampling through the dermatome from the right thigh (Fig. 2a), followed by partial thickness grafting of the penile shaft, of the scrotum, and of the perineal and suprapubic skin. During the same surgery, the left testicle was pocketed to prevent accidental trauma (Fig. 2 b – e).

**Fig. 3:**
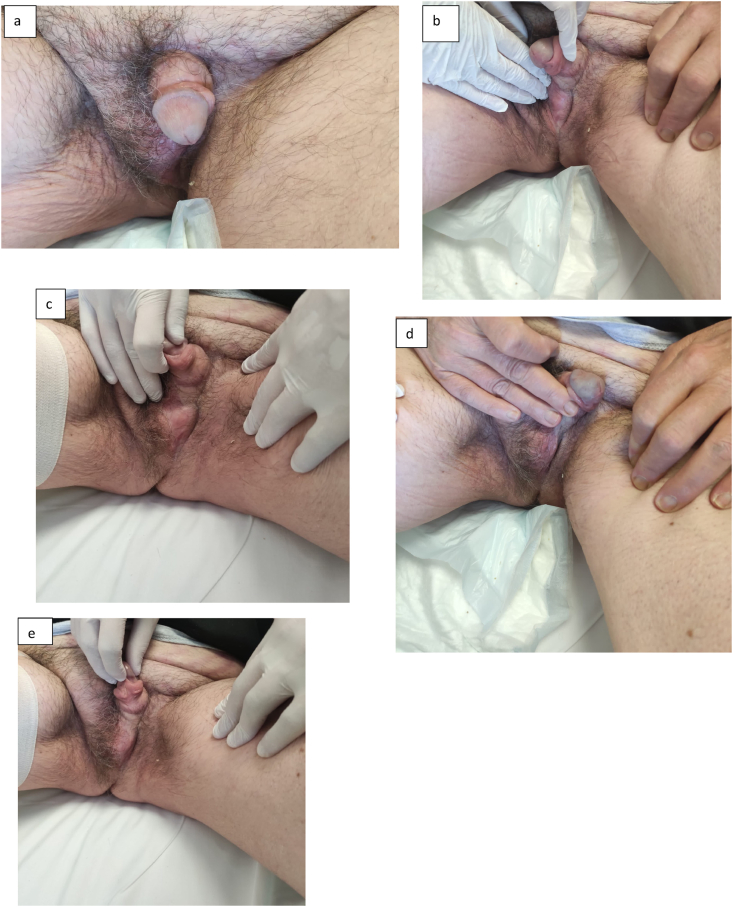
Final clinical outcome at 6 months after clinical presentation of FG.

The patient was discharged on the seventh postoperative day after the latest reconstruction surgery and was sent to a rehabilitation center for physiotherapy for 1 month (Fig. 3).

## 3 Discussion

FG is a rapidly progressive necrotizing fasciitis with a risk of sepsis in >40% of patients and has a high mortality rate of 20%–43% in contemporary series.^4^ Early diagnosis, aggressive surgical debridement, treatment of predisposing factors, and appropriate medical therapy are key to increase the patient's survival. Moreover, cooperation between anesthesiologists, urologists, plastic surgeons, infectologists, and nursing staff is necessary for a prompt diagnosis, appropriate management, and a drastic reduction in mortality.^5^ Moreover, even more important in this era of health emergency, the signs and symptoms of the patients must not be underestimated, but rather perform a precise anamnesis and visit the patient to correctly direct them towards the most appropriate therapy. Moreover, this clinical report could inspire medical staff to deliver the best patient care despite organizational difficulties.

## 4 Conclusion

It is possible to reduce the mortality of FG by making an early diagnosis, multimodal treatment, and subsequent restoration of necrotic tissues, giving the patient a satisfactory quality of life.

### Consent

Written consent was obtained from the patient and their relatives for publication of the study.

## Funding

None.

## Declaration of competing interest

None.
